# Health and healthcare provision to asylum seekers and refugees in Germany

**DOI:** 10.17886/RKI-GBE-2017-021

**Published:** 2017-03-15

**Authors:** Laura Frank, Rahsan Yesil-Jürgens, Oliver Razum, Kayvan Bozorgmehr, Liane Schenk, Andreas Gilsdorf, Alexander Rommel, Thomas Lampert

**Affiliations:** 1 Robert Koch Institute, Department Epidemiology and Health Monitoring and Department Infectious Disease Epidemiology, Berlin; 2 Bielefeld University, Faculty of Health Sciences; 3 Heidelberg University Hospital, Department of General Practice and Health Services Research; 4 Charité-Universitätsmedizin Berlin, Institute of Medical Sociology and Rehabilitation Science

**Keywords:** ASYLUM SEEKERS, REFUGEES, MEDICAL CARE, COMMUNICABLE DISEASES, NON-COMMUNICABLE DISEASES

## Abstract

The wars and devastation of recent years have driven many people to flee their homes. Great numbers of asylum seekers and refugees have sought protection in Europe. In 2015 and 2016 alone, over one million people applied for asylum in Germany. This has posed a great challenge for Germany’s healthcare provision facilities. The health of asylum seekers and refugees and the provision of their healthcare is therefore an important issue in terms of public health.

The first part of this article describes the extent and legal framework of immigration to Germany during the past two years. The second part then discusses the issue of health and medical care for asylum seekers and refugees.

Until now, no representative data on the health of this population exists. Studies so far have all relied on a small number of cases and been limited to particular regions and are therefore hard to compare. Moreover, there are no sufficiently standardised medical examinations during initial reception across all German federal states. Relevant findings suggest an urgent need to take action in the fields of mental health, chronic diseases and the provision of care to children of asylum seekers. A review of the data available proves the need for a national and systematic collection of valid data as a basis for adequate preventive and medical care. Different initiatives currently aim to improve the data collection basis in Germany. Over time, these new initiatives will significantly improve the data available on the health situation of asylum seekers and refugees in Germany. Once politics and broader society take these findings into account, this should contribute to an objective debate and evidence-based decisions.

## 1. Introduction

The number of refugees seeking protection in Germany from war, persecution and other emergencies has increased significantly in the course of 2015 and 2016. Although tighter controls at the EU’s external borders as well as the closure of the most important routes for refugees have led to a decrease in numbers, the challenges of providing adequate care to asylum seekers and refugees and ensuring their integration into society remain basically unchanged (see info box on asylum seekers and refugees). From a public health point of view, the key questions surround health and healthcare needs and whether the facilities and provision of care, as well as the legal framework conditions, can answer the specific needs of asylum seekers and refugees.

**Info box on asylum seekers and refugees**

Asylum seekers and refugees are subgroups of the population with a migrantion background (i.e. the person in question or at least one parent has immigrated to Germany). Asylum seekers is hereinafter used to designate people who reside in Germany and are currently seeking asylum or who have exceptional leave to remain in accordance with Section 60a of Germany’s Residence Act. This includes all asylum seekers, persons with exceptional leave to remain and therefore also minors. Refugee hereinafter refers to any person that according to the Geneva Refugee Convention is seeking ‘protection from persecution for reasons of race, religion, nationality, membership of a particular social group or political opinion’ (Section 3 subsection 1 of Germany’s Asylum Act).

So far, Germany, like most other EU countries, lacks the data for a clear picture of asylum seekers’ and refugees’ health and healthcare needs. Most studies to date have relied on a small number of cases and were limited to a particular region [1]. Data collection is not standardised and data is therefore hard to compare [2]. Recent initiatives, however, promise to establish a robust data basis for research and health reporting as well as political decision-making and measures. These initiatives aim for standardised data collection during initial reception examinations and gaining access to data from statutory health insurance funds. Proposals have been made to include asylum seekers and refugees, who have been in Germany for longer, in larger studies such as the Robert Koch Institute’s health survey.

This article summarises the findings available on the health situation of asylum seekers and refugees in Germany and the health care they receive. As such, it highlights current data and information gaps as well as the difficulties resulting from the legal framework conditions for the use of data and also for healthcare. To conclude, the article gives a detailed presentation of initiatives which will improve the available data. Firstly, however, the article discusses the specificities of the asylum application procedure in Germany as well as the trend in the numbers of asylum applications and refugees.

## 2. The asylum procedure and its legal framework

**Fig. 1:**
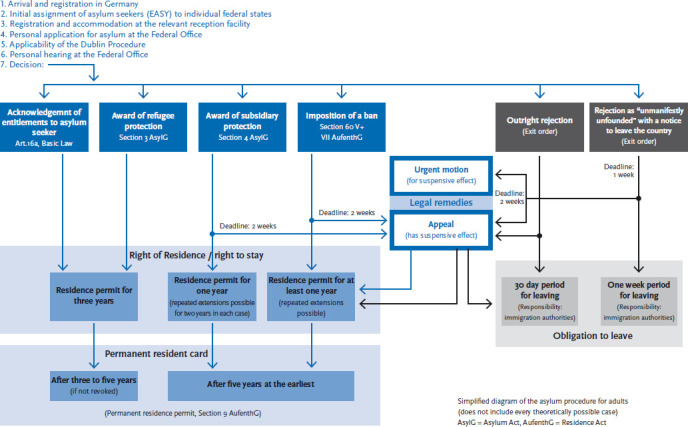
Applying for asylum in Germany Source: This figure is based on material from the Federal Office for Migration and Refugees [3, 5]

Germany’s Basic Law (Basic Law Article 16a) enshrines the fundamental right for all those suffering political persecution to apply for asylum. The country’s Asylum Act (AsylG) as shown in a simplified manner in figure 1 then regulates the actual asylum procedure. People with non-German citizenship may apply for asylum in Germany directly at the border, with the immigration authorities, the security authorities or at reception centres. They are registered and sent to the closest initial reception centre in that German federal state. The centre provides asylum accommodation and informs the closest branch of the Federal Office for Migration and Refugees. At this branch office, asylum seekers then personally apply for asylum. Before they can do so, however, the Federal Office for Migration and Refugees will determine whether that person can apply for asylum in Germany or has to do so in another country. In this case, Germany would, in accordance with the Dublin III regulation (EU regulation no. 604/2013 of the European Parliament and the Council of 26 June 2013) return the asylum seeker to that country for the asylum application to be assessed there (Dublin procedure). In most cases, this will be the country where an applicant first reached the European Union (EU). Due to the great increase in the number of asylum seekers, in particular southern European nations along the EU’s external border (Greece, Italy) however frequently no longer enforce this regulation. Only very few asylum seekers are therefore returned to other countries based on the Dublin procedure. Based on Germany’s third country regulation, which was adopted in 1993, people who enter Germany coming through other EU member states or safe third countries of origin can no longer apply for asylum in Germany on grounds of political persecution. Gaining recognition as a refugee in accordance with the Geneva Refugee Convention however remains possible.

Refugees who are minors and arrive in Germany without their parents (unaccompanied minors) seeking protection are referred to the closest youth welfare office according to Sections 42 and 42a of Germany’s Social Code, the SGB (Sozialgesetzbuch VIII), book eight, and are put in guardianship. A clearing procedure then exhaustively clarifies the situation of the unaccompanied minor. This includes verification of identity, search for family members and, where doubts exist, determining the minor’s age, assessing their overall health, and clarifying their residence status. Finally, a decision on whether or not to file an application for asylum is taken.

The Federal Office for Migration and Refugees audits all applications for asylum, calls applicants for a hearing and then takes a decision. Decisions on substance fall into one of the following four groups. A person may either be recognised as an asylum seeker according to Article 16a of the German Basic Law, as a refugee according to Section 3 subsection 1 AsylG, be granted subsidiary protection according to Section 4 subsection 1 AsylG or their application is rejected (figure 1) [3]. Recognised asylum seekers and refugees are granted a residence permit valid for three years. In most cases they will then receive permanent residency [3]. Applicants that do not obtain recognised refugee status are granted subsidiary protection if upon return to their countries of origin they would face serious threats to their well-being. These persons are awarded the right to stay in Germany for one year, a right which can be extended. In cases where it rejects an asylum application, the Federal Office for Migration and Refugees will determine whether the applicant’s life, health or freedom is threatened in his or her country of origin. If so, the office can pronounce a ban on deportation for that person in accordance with Section 60, subsections 5 or 7 of the Residence Act (AufenthG). If not, the applicant will be asked to leave the country or face deportation. If the office rejects an asylum application on grounds of considering the application unfounded or manifestly unfounded, the applicant must leave the country within thirty days or one week, respectively. The applicant can appeal against the decision of the Federal Office for Migration and Refugees. In addition to these decisions on substance, there are also decisions for formal reasons. These include Dublin procedure decisions, cases in which asylum seekers revoke their application and decisions in subsequent applications where a decision is taken to end the procedure [4]

## 3. Trend in the number of applications for asylum

According to the Federal Ministry of the Interior, 890,000 asylum seekers arrived in Germany in 2015. [6]. Estimates had reckoned with up to 1.1 million asylum seekers. The difference between these two figures results primarily from people registering twice in the system for the initial distribution of asylum seekers (EASY). EASY only registers an asylum seeker’s country of origin and destination and then anonymously distributes them between federal states. EASY does not register if a person travels or returns [6]. Meanwhile, the law on improved data exchange Germany implemented in February 2016 has reduced the risk of multiple registrations, as in most cases asylum seekers’ biometric data is registered as soon as they express their wish to apply for asylum [7].

**Fig. 2:**
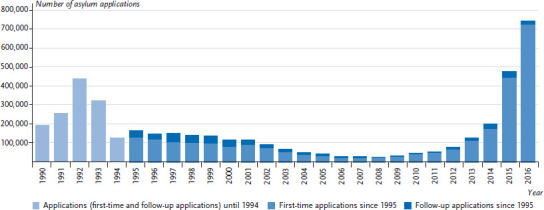
Asylum applications in Germany since 1990 Source: Federal Office for Migration and Refugees [8]

In 2015, the number of registrations in EASY was significantly higher than the total of around 480,000 asylum applications. One reason was that staff shortages at the Federal Office for Migration and Refugees led to delays in the registration and processing of asylum applications. This situation has changed greatly today. The number of asylum seekers who have recently arrived has dropped significantly, yet a high number of open cases means the number of asylum applications currently being processed remains high. In 2015, the Federal Office processed 476,649 applications for asylum. 441,899 were first-time applications and 34,750 follow-up applications [4]. Compared to year before, the number of applications rose by 155.3%. In 2016, there were 745,545 applications. Of these, 722,370 were first-time applications and 23,175 follow-up applications [8]. Compared to 2015, this implies a 56.4 percent increase in asylum applications. At the end of December 2016, 433,719 applications for asylum remained pending at the Federal Office for Migration and Refugees [9]. Figure 2 shows the long-term trend in the number of applications. Following a peak in 1992, the number of applications dropped significantly between 1993 and 2008 in the wake of the introduction of the so-called third country regulation. Since then, the annual number of asylum applications has picked up again.

**Fig. 3:**
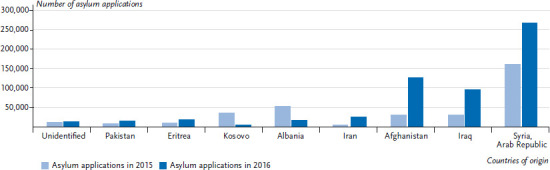
Asylum applications from top countries of origin in 2015 and 2016 Source: Federal Office for Migration and Refugees [4, 9]

In 2015 and 2016, Syria, Afghanistan and Iraq were the most important countries of immigration (figure 3). In 2016, three quarters of all asylum applications came from one of these three countries (Syria, 36.9%; Afghanistan, 17.6%; Iraq, 13.3%) [8]. A clear drop in the number of applications was registered in 2015 and 2016 for people from Albania and Kosovo, which were declared safe countries of origin in autumn 2015 [10].

In 2015 (69.2%) [4] and 2016 (65.7%) [8], the majority of asylum seekers was male. This gender distribution ruled across all age groups with the exception of those aged “sixty-five and older” where the number of female applicants was greater (2015: 53.4%, 2016: 54.2%). The majority of applicants was under thirty (2015: 71.1%, 2016: 73.8%) and a third of these asylum seekers was aged under eighteen [4, 8].

In particular, the number of applications for asylum by unaccompanied minors (14,439 applications) increased markedly in 2015 and more than tripled compared to 2014. In 2015, the majority of unaccompanied minors came from Afghanistan (32.9%), followed by Syrians (27.6%), Eritreans (9.3%) and Iraqis (9.3%) [4]. The trend for 2016 points to a further increase in first-time applications for asylum by unaccompanied minors to 35,939 [11].

### 3.1 Decisions on asylum applications by the Federal Office for Migration and Refugees in 2015 and 2016

The following overview shows the decisions and decision rates regarding asylum applications taken by the Federal Office for Migration and Refugees for 2015 and 2016 (table 1). If one relates the number of positive decisions e.g. that led to some form of residence permit (recognition as asylum seeker or refugee, subsidiary protection and/or ban on deportation) to the total number of decisions during that same period, then the result is a total protection rate [4]. In 2015, the Office decided on 282,726 asylum applications, leading to a total protection rate of 49.8% [12]. In comparison, in 2016, the Federal Office decided on a total number of 695,733 asylum applications, resulting in a total protection rate of 62.4% [8]. Total protection rates vary greatly for different countries of origin. For people from Syria, it was 98% in 2016; for people from Albania, however, only 0.4% [9].

The majority of successful asylum applications lead people to be awarded the status of recognised refugee. Only a small number of applicants is awarded political asylum according to Germany’s Basic Law. Out of the total number of asylum seekers whose applications were decided in 2015, 48.5% received refugee status (table 1). Only 0.7% received the legal status as entitled to asylum. For 2016, the corresponding figures were 36.8% and 0.3%, respectively. However, the share of people receiving subsidiary protection increased to 22.1% in 2016 (2015: 0.6%) [8].

### 3.2 The distribution of asylum seekers in Germany

To a certain degree, the distribution of asylum seekers to initial reception centres in the federal states depends on accommodation capacities. Furthermore, the local branches of the Federal Office for Migration and Refugees are each responsible for particular countries of origin which means that the distribution of asylum seekers also depends on the state where a particular branch is located. Moreover, there are quotas for each federal state. These are calculated according to the Königsteiner key (Section 45 AsylG). The key regulates the distribution of asylum seekers to initial reception centres in the federal states. The Königsteiner key is based on a federal state’s annual tax revenue and population. Since 1 November 2015, unaccompanied minors are also distributed across Germany using the Königsteiner key (in accordance with Sections 42c and 42d SGB VIII) [13]. Initially, unaccompanied minors are referred to local youth welfare offices. Later, the federal and state authorities seek accommodation for these minors near the relevant youth welfare office. If a state has already fulfilled its quota, the unaccompanied minor is brought to the closest possible state.

**Fig. 4:**
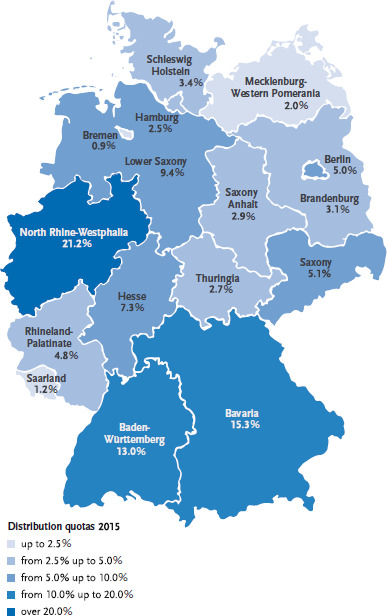
Distribution quotas of asylum seekers based on the Königsteiner key in 2015 Source: Federal Office for Migration and Refugees [4]

Figure 4 shows the distribution of asylum seekers based on the Königsteiner key for 2015. Distribution was proportional to the population of individual federal states. North Rhine-Westphalia therefore received the largest share of applicants (21.2%) and Bremen the smallest (0.9%). The distribution policy does not, however, take into account the different health and social needs of asylum seekers. A high number of people from particularly vulnerable groups such as children, the elderly and women with special needs are assigned to states with low tax revenue such as Bremen, Berlin and North Rhine-Westphalia [14].

## 4. Current research and findings on asylum seeker and refugee health

Representative data on the health and healthcare of asylum seekers and refugees in Germany is still lacking [2]. Compulsory examinations in accordance with Section 62 AsylG and Section 36 Infection Protection Act (IfSG) before or immediately after being admitted to an initial reception centre could prove a valuable source of data. These examinations serve to detect communicable diseases such as contagious pulmonary tuberculosis. The Robert Koch Institute has developed recommendations for minimum standards for standardised initial reception examinations in accordance with Germany’s asylum law [15]. Centres should ensure pulmonary tuberculosis screening across Germany; further examinations are at the discretion of the concerned federal state’s public health authorities. The scope and content of screening examinations therefore vary considerably between federal states [16], data is hard to compare and cannot be analysed across Germany [2]. The following picture of the health of asylum seekers and refugees can therefore only build on limited data.

### 4.1 Communicable diseases

Asylum seekers face the same communicable disease risks as any other people in Germany. Harsh living conditions during flight, possibly only partial immunisation, higher prevalence in the countries of origin and the cramped conditions of mass accommodation increase the vulnerability of asylum seekers to communicable diseases. Currently, the Robert Koch Institute cannot confirm that asylum seekers increase the risk of contracting communicable diseases amongst the general public [17]. The potential for outbreaks of communicable diseases, however, is a great concern in initial reception centres. An analysis of the notifications on outbreaks of communicable diseases between 2004 and 2014 in accommodation centres for asylum seekers collected based on the Infection Protection Act shows an increase in cases [18]. Data also showed that most of those sick had contracted their condition whilst being in Germany. During the period, centres notified the Robert Koch Institute in accordance with the Infection Protection Act of 119 outbreaks involving 615 individual cases in asylum seeker accommodation centres. The most frequent disease was chickenpox (29% of cases), followed by scabies (18% of cases), measles (12% of cases), tuberculosis and rotavirus gastroenteritis (8% of cases), and other diseases (less than 5% of cases). Rarely did outbreaks spread beyond the boundaries of accommodation centres. Early vaccination, provision of information and better hygiene would have prevented most of these outbreaks [18].

Since the end of 2015, based on the data it receives according to the Infection Protection Act, the Robert Koch Institute has prepared a weekly report on the spread of communicable diseases amongst asylum seekers [19]. From calendar week 1 to calendar week 52 in 2016 (based on the data available on 18 January 2017), 6,326 cases were transmitted amongst asylum seekers. This is 1.9% of all cases of notifiable communicable diseases (329,974) in the population. Since early 2016, a small decline in the number of cases has been registered. Data on the current number of asylum seekers and their geographic distribution across Germany is incomplete, which makes it difficult to relate this figure to the ultimate number of notifications across the population. Moreover, no systematic screening for communicable diseases in the general population takes place. This makes it hard to compare incidence and prevalence among asylum seekers to other sections of the population.

Prevalence of particular communicable diseases is higher among asylum seekers than among the resident population. One good example is hepatitis B (HBV). Prevalence of the HBV surface antigen (HBsAg) as the most important indicator for a hepatitis B infection was significantly higher among asylum seekers arriving in Germany (2.3%) than among the overall population [20]. Screening programmes among asylum seekers have led to an increased detection of tuberculosis and hepatitis B and C. Only isolated cases of other imported severe communicable diseases such as relapsing fever have so far been reported [17].

With the exception of compulsory pulmonary tuberculosis examinations, screening programmes differ considerably from one federal state to the next [16, 21], leading to disparities in the prevalence of communicable diseases between different studies. A study of 102 unaccompanied minors aged 12-18 years in Bielefeld reported the high prevalence (58.8%) of communicable diseases. This was mainly due to the high rates of helicobacter pylori infections. Tuberculosis prevalence was about 1% [22]. However, the Bremen health programme reported low prevalence of communicable diseases among asylum seekers between 2001 and 2008 [23] as well as for 2011 to 2014 [24].

Gastrointestinal infections and vaccination-preventable diseases continue to be a focus. Studies reveal that asylum seeker immunisation is rarely surveyed consistently [21] and infants in particular are often only partially immunised [25]. The Standing Committee on Vaccination (STIKO) therefore recommends rapidly immunising all asylum seekers with only partial or unknown immunisation status [26]. Information on vaccination is currently made available in 19 languages [27]. On its website, the Robert Koch Institute provides an overview of epidemiologically relevant communicable diseases [28] and vaccination recommendations [19].

However, clear difficulties in the implementation of STIKO recommendations remain, for example concerning polio vaccination and stool screening for poliovirus following the 2013 outbreak of polio in Syria [29]. An analysis of the implementation of these recommendations by initial reception centres revealed significant differences [30]. Difficulties with implementation increased relative to the size of a centre. Centres pointed to staff shortages and language barriers as the greatest obstacles to implementation [30].

### 4.2 Non-communicable diseases

Non-communicable diseases such as diabetes mellitus, cardiovascular diseases, cancer, chronic respiratory diseases and mental disorders imply a high disease burden for the German population. Studies on non-communicable diseases among asylum seekers in Germany have focussed mainly on mental disorders [1].

#### Mental disorders

Some refugees and asylum seekers coming from regions torn by conflict or war have experienced warfare, political persecution, torture, attacks and sexual assaults before fleeing their home countries. Added to these traumatic experiences in their countries of origin are the ordeals of their long flight. Asylum seekers and refugees frequently come through various transit countries, which means their flight often takes months or even years. During this time, they not only fear for their own lives, many also lose relatives or witness the death of other refugees. Unaccompanied minors are particularly vulnerable to attacks and sexual assaults during flight [31].

Traumatic experiences can increase the risk of post-traumatic disorders [32] such as post-traumatic stress disorders, depressions, anxiety disorders, chronic pain and somatoform disorders. Post-migration stressors such as hearings, the duration of the asylum procedure, separation from closest attachment figures as well as discrimination and language difficulties are also risk factors for mental disorders [32].

Data for 104 accompanied minors from thirteen accommodation centres in Baden-Württemberg shows a high prevalence of traumatic incidents: 41.3% of these minors had witnessed physical attacks, 37.5% military conflicts, 25.0% had seen dead bodies, 15.4% had been personally physically attacked and 4.8% had been sexually abused [33]. Compared to accompanied minors, unaccompanied minors are roughly twice as likely to experience traumatic incidents. According to a study from the Netherlands, out of 1,187 accompanied minors, 23.2% had reported being physically and 8.3% sexually abused. For 1,100 unaccompanied minors, these rates were significantly higher (63.3% and 20.3% respectively). 39.3% of female and 12.1% of male unaccompanied minors had suffered sexual abuse [34].

The prevalence of post-traumatic stress disorders among accompanied and unaccompanied minors in Germany was between 14.0 and 60.0% [35, 36] and for depressions between 6.2 and 33.5% [35]. A systematic overview of mental disorders among asylum seekers (minors and adults) shows that prevalence estimates for post-traumatic stress disorders vary widely both in institution-based samples (6.7-76.7%) and in population-based samples (16.4-54.9%) [1]. The wide divergence in prevalence has various reasons. The number of cases and methods of selecting a sample play an important role as does the composition of the sample. In addition, the heterogeneity of countries of origin and cultural backgrounds as well as the methods and measuring instruments used influence the diagnosis of post-traumatic stress disorders [1]. Especially culturally or linguistically not adapted screening and diagnostic instruments are a particularly challenging factor in detecting post-traumatic mental disorders [35]. The possibility of misdiagnoses cannot be ruled out. Prevalence among asylum seekers is however considerably higher than among the general population in Germany. Representative surveys show a lifetime prevalence of post-traumatic stress disorders among children and adolescents in Germany of 1.3% [37] and 5.4% for depressions [38].

Additionally, many asylum seekers and refugees suffer from conditions such as back pain, headaches or neck pain, which often occur together with post-traumatic stress disorders [39]. Doctors diagnosed these conditions among 16.9% [24] and 25.4% [23] of patients respectively in the accommodation centres of the Bremen health programme (Bremer Modell). This could indicate high levels of mental stress [24] and be a reaction to the stress of flight, arrival in Germany and the stress related to accommodation and the uncertainty of their prospects to settle in the country.

#### Further non-communicable diseases

Data on other non-communicable chronic diseases such as cancer, chronic respiratory diseases, diabetes mellitus and cardiovascular diseases also remains fragmentary [1]. One reason is that currently mainly young people (under thirty) who seldom suffer from chronic diseases are applying for asylum in Germany. A study from Bielefeld revealed the low prevalence of non-communicable chronic diseases among 102 unaccompanied minors. Asthma prevalence was 3.9%, two minors were diagnosed with lipo-metabolic disorders and severe obesity, one with an infection of the bone marrow (osteomyelitis) and one with post-polio syndrome [22].

Medical practices that migrants and refugees in the accommodation centres in Bremen can turn to on a voluntary basis based on the Bremer Modell provide data on various diseases among asylum seekers for the analysis period 2011-2014 [24]. In 29.6% of cases, ‘Factors for seeking healthcare’ were the most common reason for seeking medical consultation. These include initial medical examinations at reception without confirming a disease, provision of information on vaccination and counselling of pregnant women. Respiratory diseases were among the most frequent ICD-10 (International Classification of Diseases, 10th revision) diagnoses (18.1% of cases). Largely, this concerned acute colds that asylum seekers contracted mostly in accommodation centres. Secondly came unclear symptoms, not classified elsewhere, in particular unspecific pains without identifiable organic causes such as headaches (16.9% of cases). Thirdly came diseases of the digestive system (6.1%), which can be related to unfamiliar foods and irregular meals as well as psychosomatic causes due to mental stress [24]. Further diagnoses included musculoskeletal and connective tissue diseases (each 6.0%) as well as diseases of the skin and hypodermis (3.6%). Acute dental problems, which were not treated before or during flight, showed a strong link to patients’ country of origin [24]. Approximately twenty percent of the 102 unaccompanied minors in Bielefeld showed tooth pathologies [22, 36]. In addition, iron-deficiency anaemia was far more frequent among unaccompanied female minors (29.2%) than unaccompanied male minors (14.1%) [22].

## 5. Healthcare and access to the healthcare system

Providing continuous healthcare to sick asylum seekers creates specific challenges for the German healthcare system [20]. A survey of all public health authorities revealed that interviewees felt that levels of care were inadequate for asylum seekers in Germany with mental illnesses or severe chronic diseases. Healthcare provision to the children of asylum seekers was also not evaluated as being ensured to the same degree as for unaccompanied minors [21].

Depending on federal state access to medical care is restricted to varying degrees during the first fifteen months of stay. Many stakeholders believe that this means that asylum seekers and refugees cannot expect adequate levels of care across Germany [40-42]. The text discusses the legal entitlement to healthcare, access to the German healthcare system and further related problems in more detail below.

### 5.1 Healthcare and the Asylum Seekers’ Benefits Act

The Asylum Seekers’ Benefits Act (AsylbLG) regulates healthcare for asylum seekers. According to Section 4 subsection 1, asylum seekers suffering from acute, treatable diseases and pain are entitled to receive healthcare services. In these cases, according to the law, ‘necessary medical or dental treatment has to be provided including medication, bandages and other services necessary for convalescence, recovery, or alleviation of disease or consequences of illnesses. Dental prostheses are only provided where for medical reasons such a measure cannot be delayed.’

Chronic conditions liable to turn acute without treatment or to lead a patient’s health to deteriorate may also be treated under the Asylum Seekers’ Benefits Act. Preventive medical check-ups and vaccinations are to be granted in every case. Pregnant women are entitled to medical and nursing care and support, midwife assistance, medications, bandages and remedies pursuant to Section 4 subsection 2 AsylbLG. All asylum seekers should be offered full immunisation early upon arrival (pursuant to Section 4 subsection 3 AsylbLG).

In addition, the law states that further benefits can be granted ‘if they are […] essential in an individual case to secure life or health’ (Section 6 subsection 1 AsylbLG). Furthermore, ‘persons who acquire a residence permit pursuant to Section 24 subsection 1 of the Residence Act (Aufenthaltsgesetz) and have special needs such as unaccompanied minors or those who have suffered torture, rape or other severe forms of physical, mental or sexual violence shall receive the medical services and other assistance they require’ (Section 6 subsection 2)

Many criticise the restrictions Sections 4 and 6 AsylbLG impose on the right of asylum seekers to receive treatment in case of illness relative to people covered by normal statutory health insurance and the implementation of these restrictions in practice [41-43]. An important point of criticism is that asylum seekers are not granted electronic patient cards. Every time they wish to see a doctor, they must apply for a voucher for medical treatment in advance from the competent authority, for example at their social welfare office. In many cases, people without the necessary medical expertise then decide on these voucher applications [41].

### 5.2 Healthcare on the basis of electronic health cards

After at most fifteen months, asylum seekers are entitled to full healthcare services and receive an electronic health card from a statutory health insurance scheme in accordance with book twelve of the Social Code (SGB) [40]. Nonetheless, they still do not have the same status as regular statutory health insurance members because the costs of treatment, plus a five percent administration fee, are reimbursed to insurers by social welfare offices in accordance with Section 264 of book five SGB [40].

In Germany, access to the healthcare system is not regulated at national level. In some states, asylum seekers will receive an electronic health card even before fifteen months. Since 2005, Bremen, for example, has granted such cards around three months after initial registration [23]. With few exceptions, healthcare includes the same services as those offered to regular members of statutory healthcare insurance schemes. Among the services not included are fertility treatments, disease management programmes, child benefit and maternity allowance, and medical services contracted outside of Germany. Moreover, asylum seekers will generally not have access to psychotherapy, preventive and rehabilitative care, visual aids, dental prostheses and orthodontics [44], but may be granted these after assessment in individual cases. In 2012, Hamburg also introduced the electronic health card for asylum seekers. The city has excluded services such as long-term psychotherapies, rehabilitation therapy, dental prostheses and contracting services outside of Germany [40].

In October 2015, Germany adopted the Asylverfahrensbeschleunigungsgesetz, a law to fast-track asylum applications. It makes obtaining an electronic health card easier for asylum seekers even before they have been in Germany for fifteen months. Each federal state can decide whether to apply the law or maintain the existing structures. Statutory health insurers are then required to conclude a framework agreement with that state. A pre-condition is that the agreement will apply at least at district level or for independent towns [40]. Municipalities are free to opt in to these agreements. Framework agreements define the scope of services, accounting procedures and accounting audits for services as well as the reimbursement of the costs of care and for administration incurred by insurers [40].

Since adoption of the law, Schleswig Holstein and Berlin have introduced electronic health cards for all asylum seekers, considerably lowering the barriers to the healthcare system. In North Rhine-Westphalia, 20 municipalities have so far adopted a framework agreement between the federal state and municipalities. However, services remain based on Sections 4 and 6 AsylbLG, implying that asylum seekers in these states have only limited access to medical care compared to regular members of statutory healthcare [40]. Lower Saxony, the Rhineland Palatinate, Saarland, Brandenburg and Thuringia have all concluded framework agreements with statutory health insurers. Many municipalities have however not yet entered these agreements. Hesse is still negotiating with insurers and municipal umbrella organisations [45]. Bavaria, Saxony, Baden Württemberg, Mecklenburg-Western Pomerania and Saxony-Anhalt decided not to introduce an electronic health card for asylum seekers [45].

As the available data reveals, limiting access to medical services for asylum seekers hardly helps save costs. Indeed, data from the Office for Labour, Social Affairs, Family and Integration in Hamburg indicates that equipping asylum seekers with electronic health cards could help save costs. While the average per person costs of healthcare remain the same, considerable amounts are saved that would otherwise have been spent on administrative fees [46]. A study based on data from the Federal Statistics Office of the period from 1994 to 2013 reveals that annual per capita medical services expenditure for asylum seekers with only limited access to the healthcare system are higher than for asylum seekers with full access [47]. There are, therefore, good arguments to reduce the barriers to healthcare services from statutory health insurers both from a humanitarian and an economic point of view.

### 5.3 Psychiatric and psychotherapeutic care

In acute cases, the Asylum Seekers’ Benefits Act also covers psychiatric care. Psychotherapy, however, is generally excluded. Psychotherapy is therefore often applied for based on Section 6 AsylbLG, which provides the possibility of at least short-term therapy. A further obstacle for psychotherapy concerns the reimbursement of the costs of interpretation during therapy sessions [32]. According to Sections 4 and 6 AsylblG, asylum seekers still awaiting the decision over their asylum application can apply to have the costs for interpretation reimbursed from their social welfare office [48]. Minors stand the greatest chances of having these costs covered. Recognised asylum seekers and asylum seekers who have been in Germany for more than fifteen months, however, will find getting reimbursement for interpretation hard. As Germany’s Social Code books define German as the country’s official language, statutory health insurers will not cover these costs. Reimbursement for interpretation can however be applied for according to Section 73 book twelve SGB or in certain cases according to Section 53 book twelve SGB from the social welfare offices; yet these constitute discretionary benefits [35]. The lack of regulation concerning reimbursement for interpretation therefore constitutes a major barrier for access to psychotherapy [32, 35].

Psychotherapeutic treatment in Germany remains organised in specialised psychosocial treatment centres. The umbrella organisation of psychosocial centres for refugees and the victims of torture BAfF (Bundesweite Arbeitsgemeinschaft der psychosozialen Zentren für Flüchtlinge und Folteropfer) is a network of 26 psychosocial centres in Germany [49]. One quarter of patients at these centres are minors and of these approximately 45% are unaccompanied minors [49]. Limited provision and difficult access to psychotherapeutic and psychosocial facilities prevents the early treatment of often severely traumatised patients. Only very few asylum seekers therefore access therapy. According to BAfF, social security offices rejected 15% of all applications for psychotherapy. In contrast, only 1 to 3% of applications by members of statutory health insurers are rejected [49].

Moreover, centres registered an imbalance between supply and demand. Each year they turn down considerably more asylum seekers than they can treat. This leads to long waiting lists and psychosocial centres only refer a mere 5% of patients to registered therapists each year [49]. In addition, early detection of asylum seekers who are under considerable mental strain and therefore at increased risk of becoming ill would require new linguistically and culturally sensitive instruments for screening and diagnosis [35]. Furthermore, there is an increasing need for professionals specially qualified and trained in psychotherapy for children and adolescents [35]. A multi-modal concept supported by interpreters that combines psychotherapy with social work, medical care and legal counselling on residence is recommended for the treatment of traumatised asylum seekers [32].

## 6. Discussion

The strong rise in the number of asylum seekers poses significant challenges to the German healthcare system. Initial results indicate a particular need for care concerning mental health and chronic diseases as well as for the children of asylum seekers [1, 21]. Many stakeholders therefore uphold the need to grant asylum seekers the same rights to access healthcare services as everybody else as early as possible [41, 50]. Furthermore, in the relevant literature we can identify a number of areas that many stakeholders believe require action:

► Experts broadly agree on the need to rapidly implement the recommendations of the Standing Committee on Vaccination for asylum seekers [18, 21, 26, 30]. In large reception centres, staff shortages and language barriers appear to be potential obstacles [30].
► Asylum seekers appear to have an increased need for information on issues such as vaccination and pregnancy [24]. For public health authorities, important information includes materials that provide orientation in the healthcare system, information on local contact points for health questions, vaccine-preventable diseases, sexually transmitted diseases, diet during pregnancy and shortly after birth, dental hygiene for children, mental disorders, addictive behaviour as well as tuberculosis [21].
► Mental health is a field many observers consider requires more action [31, 32, 35, 51]. Very great needs, they believe, meet with poorly developed care facilities. The lack of validated screening instruments capable of taking into account language and culture is problematic [35]. Moreover, AsylbLG limits access to therapy. The lack of qualified therapists, overstrained care facilities as well as the unclear reimbursement of services such as interpretation remain obstacles even once therapies have been granted [32, 35, 51].
► Many experts moreover recommend providing asylum seekers with an electronic health card [41, 52]. This would help reduce administration costs and would lower the barriers for asylum seekers to access healthcare services [41]. A crucial advantage would be that asylum seekers would no longer have to apply for vouchers at social security offices in advance and doctors could decide treatments directly [41].

Recognising gaps in healthcare and care needs requires reliable information. Studies so far have frequently relied on low case numbers, been limited to certain regions and are hardly reliable [1]. Results cannot be compared due to the highly heterogeneous nature of study populations and survey instruments [1]. A lack of comparative figures for the general population also makes the classification of the health problems of asylum seekers and refugees more difficult. Generally, the data available is not sufficiently differentiated either. Asylum seekers are a highly heterogeneous population with different resources, health problems and medical needs. Current examinations do not usually differentiate between women and men, which has so far hampered a gender-sensitive analysis. Due to the great heterogeneity of this group, an approach would be desirable that differentiates between people according to their country of origin, cultural background or possible reasons for flight for example.

Health examination procedures in initial reception centres are, however, also responsible for the current lack of reliable information [2, 21]. Relevant information is not always collected and transmitted as it should be [21]. Furthermore, different federal states demand different examinations [16].

Recently, therefore, important initiatives have developed to improve information on the health of asylum seekers and refugees and promote close networking between important stakeholders. RESPOND, a project by the University Hospital of Heidelberg, which is funded by the Federal Office of Education and Research, has begun population surveys on the health and healthcare of asylum seekers in Baden Württemberg [53]. The Federal Ministry of Health also funds another initiative by the Heidelberg University Hospital to collect data on the health and healthcare of asylum seekers and refugees (Dateninitiative Gesundheit und medizinische Versorgung von Asylsuchenden und Flüchtlingen) which is considering options to standardise documentation on medical care at reception centres in the federal states [54]. The initiative aims for a rapid detection of health problems and the use of information for routine reporting and scientific analysis. The research group FlüGe – Herausforderungen und Chancen globaler Flüchtlingsmigration für die Gesundheitsversorgung in Deutschland (FlüGe – Opportunities and threats of global refugee migration to healthcare in Germany) at the University of Bielefeld is also investigating specific issues related to the health situation and healthcare of asylum seekers and refugees [55]. North Rhine-Westphalia’s Ministry for Innovation, Science and Research is funding two further projects at the University of Bielefeld to investigate the effects of providing asylum seekers with electronic health cards.

Moreover, the Robert Koch Institute is carrying out various projects in the context of infection protection. For example, syndromic surveillance in emergency shelters for asylum seekers in Berlin and the evaluation of data provided by medical care units improves treatment. Furthermore, knowledge on the prevalence of multi-resistant pathogens as well as on hepatitis and tuberculosisis improved, creating a better basis to develop recommendations. In times of increased immigration and to fulfil the information requirements laid out in the Infection Protection Act earlier and better in the future, Germany has updated reporting by introducing DEMIS (Deutsches Elektronisches Meldesystem für den Infektionsschutz) – an electronic reporting system for infectious diseases.

Asylum seekers and refugees are not however the only focus of the initiatives at the Robert Koch Institute. Gaps remain in the information concerning migrants who have been living in Germany for a long time and the children of migrants who were born in Germany. For example, as part of the Robert Koch Institute’s representative population health surveys, the baseline of the German Health Interview and Examination Survey for Children and Adolescents (KiGGS baseline survey, 2003-2006) included a proportion of children and adolescents with migration background [56]. This however does not apply to the follow-up survey KiGGS Wave 1 (2009-2012), nor to the German Health Update (GEDA) surveys and the German Health Interview and Examination Survey for Adults (DEGS1) [57].

The project funded by the Federal Ministry of Health is therefore seeking measures to improve the information base on the health of people with migration background, which aim to advance health monitoring and reporting at the Robert Koch Institute. Firstly, access methods, recruiting procedures and also the content and survey instruments will be reviewed and further developed to improve the integration of people with migration background in health monitoring. Secondly, possibilities of using routine data and micro census data as well as a socio-economic panel will be considered.

In future, these initiatives will significantly improve the information base on the health of asylum seekers and people with migration background in Germany. Based on existing experience [56, 58, 59], federal health reporting will provide important groundwork to develop a reporting concept that specifies key issues and sources of data, ensures findings are transmitted to politics and society and serves as a basis for debate in society and policy initiatives.

## Key statements

- Asylum seekers face the same communicable diseases as the general population.
- Access to healthcare services in Germany is regulated at federal state level.
- To ensure the provision of adequate healthcare services, early and easy access to the healthcare system should be considered.
- Unspecific regulations for the reimbursement of interpretation effectively bar people’s access to psychotherapy.
- There is a priority need for care in mental health, chronic disease and children.
- Robust information requires improving data on the health situation and healthcare needs of asylum seekers.

## Figures and Tables

**Table 1:** Decisions and decision rates for asylum applications in 2015 and 2016 Source: Federal Office for Migration and Refugees [8]

Year	2015		2016	
	Number	Percentage	Number	Percentage
**Total number of decisions on asylum applications**	282,726		695,733	
**Total protection rate**	140,915	49.8 %	433,920	62.4 %
**Substantive decisions**				
Legal status as refugee (Section 3 subsection 1 AsylG, Art.16a, Basic Law)	137,136	48.5 %	256,136	36.8%
of those recognised as entitled to asylum (Art. 16 a Basic Law and family asylum)	2,029	0.7 %	2,120	0.3 %
Subsidiary protection (Section 4 subsection 1 AsylG)	1,707	0.6 %	153,700	22.1 %
Ban on deportation (Section 60 subsections 5 or 7 AufenthG)	2,072	0.7 %	24,084	3.5 %
Rejections (unfounded or manifestly unfounded)	91,514	32.4 %	173,846	25.0 %
**Formal decisions**	50,297	17.8 %	87,967	12.6 %
